# A multiplex financial network approach to policy evaluation: the case of euro area Quantitative Easing

**DOI:** 10.1007/s41109-018-0098-8

**Published:** 2018-11-19

**Authors:** Chiara Perillo, Stefano Battiston

**Affiliations:** FINEXUS Center for Financial Networks and Sustainability, Department of Banking and Finance, University of Zurich, Zurich, Switzerland

**Keywords:** Unconventional monetary policy, Expanded Asset Purchase Programme, Quantitative Easing, Real economy, Financial stability, Multilayer network, Macro-network, Financial network

## Abstract

Over the last decades, both advanced and emerging economies have experienced a

striking increase in the intra-financial activity across different asset classes and increasingly complex contract types, leading to a far more complex financial system. Until the 2007-2008 crisis, the increased financial intensity and complexity was believed beneficial in making the financial system more resilient and less vulnerable to shocks. However, in 2007-2008, the advanced economies suffered the biggest financial crisis since the 1930s, followed by a severe post-crisis recession, questioning the adequacy of traditional tools in predicting, explaining, and responding to periods of financial distress. In particular, the effect of complex interconnections among financial actors on financial stability has been widely acknowledged. A recent debate focused on the effects of unconventional policies aimed at achieving both price and financial stability. Among these unconventional policies, Quantitative Easing (QE, i.e., the large-scale asset purchase programme conducted by a central bank upon the creation of new money) has been recently implemented by the European Central Bank (ECB). In this context, two questions deserve more attention in the literature. First, to what extent, the resources provided to the banking system through QE are transmitted to the real economy. Second, to what extent, the QE may also alter the pattern of intra-financial exposures and what are the implications in terms of financial stability. Here, we address these two questions by developing a methodology to map the multilayer macro-network of financial exposures among institutional sectors across financial instruments (i.e., loans and deposits, debt securities, and equity), and we illustrate our approach on recently available data. We then test the effect of the implementation of ECB’s QE on the time evolution of the financial linkages in the multilayer macro-network of the euro area, as well as the effect on macroeconomic variables, such as consumption, investment, unemployment, growth, and inflation.

**Electronic supplementary material:**

The online version of this article (10.1007/s41109-018-0098-8) contains supplementary material, which is available to authorized users.

## Introduction

Over the last decades, both advanced and emerging economies have experienced a striking increase in the financial interactions among the different actors operating in the global economy, via different financial instruments. A large part of these interactions is intra-financial (D’Errico and Roukny 2017), meaning that a great portion of banks’ financial contracts has as a counterparty another bank or another financial institution (such as investment funds, insurance corporations, and pension funds). As a consequence, a large fraction of financial system’s assets is intra-financial (Allahrakha et al. 2015). This growth in the financial activity was spread across different asset classes and contract types and went along a deep transformation of the intermediation system that connects end borrowers with end savers (Turner 2017). In particular, this transformation was based on complex innovations of existing financial instruments leading to the emergence of complex financial activities (such as credit securitization^1^ and credit structuring^2^), as well as the origin of new complex financial instruments (such as Collateralized Loan Obligations^3^, Collateralized Debt Obligations^4^, and Credit Default Swaps^5^) and, therefore, to a far more complex financial system (Turner 2017).

Until the 2007-2008 crisis, based on the assumption that the rise in the financial interactions (mainly due to the increased intra-financial interactions (Wyman 2010)) was allowing for risk diversification, the increased financial intensity and complexity was believed helpful in making the financial system more resilient and less vulnerable to shocks (International Monetary Fund (IMF) 2006). However, in 2007-2008, the advanced economies suffered the biggest financial crisis since the 1930s, followed by a severe post-crisis recession, questioning the adequacy of traditional tools in predicting, explaining, and responding to periods of financial distress. More specifically, on the one hand, the global financial crisis has shown that intra-financial linkages represent a mechanism for the propagation of financial distress and they may lead to the amplification of small shocks (Bardoscia et al. 2015; Visentin et al. 2016). Consequently, intra-financial interconnectedness is nowadays recognized as one of the key elements of potential financial instability or systemic risk (i.e., the risk of the default of a large portion of the financial system) (Battiston et al. 2016; Roukny et al. 2018). On the other hand, the long-lasting socio-economic impact of the global financial crisis has been the main impetus for a conversation about the adequacy of the existing policy responses to the crisis. In particular, a recent debate focused on the adoption, implementation and effectiveness of unconventional policy tools, aimed at achieving both price and financial stability, and able to account for the complex intra-financial interconnections that could affect financial stability (Balasubramanyan and VanHoose 2013; Claessens et al. 2013; Miles et al. 2013; Aiyar et al. 2014; Popoyan et al. 2017). Among these unconventional policy tools, the expanded Asset Purchase Programme (APP), has been recently implemented by the European Central Bank (ECB). This large-scale asset purchase programme encompasses the set of measures commonly known as Quantitative Easing (QE). Indeed, the APP includes all purchase programmes under which private and public securities (such as sovereign and corporate bonds, and asset-backed securities) are purchased by the Eurosystem from the banking system on the secondary market, in order to address the risk of a too long period of low inflation^6^ (European Central Bank 2017a). Monthly net purchases of public and private sector securities amounted to 60 and 80 billion euros on average from March 2015 to December 2017^7^. They are intended to be carried out, at a reduced monthly pace of 30 billion euros since January 2018, until September 2018, conditionally to an adjustment in the path of inflation consistent with the aim of achieving an inflation rate close to the 2% over the medium term (European Central Bank 2017a). The large-scale purchases imply the increase in the prices of the purchased assets and the provision of liquidity to the banking system. As a consequence, a wide range of interest rates falls and loans become cheaper (European Central Bank 2017c). Therefore, on the credit demand side, firms and households may be able to borrow more and spend less to repay their debts, while, on the credit supply side, the availability of new liquidity may stimulate bank lending activity. If so, consumption and investment may receive a boost and support economic growth and job creation and, as prices rise, the ECB achieves an inflation rate below, but close to, 2% over the medium term (European Central Bank 2017c). Consequently, according to the ECB’s narrative, QE may generate the increase in bank credit to the real economy and all the subsequent aforementioned chain of effects. However, QE does not necessarily imply that the resources received by banks are going to be lent to the real economy. In fact, on the one hand, businesses and households could be not willing or able to borrow more from banks. On the other hand, banks could either decide to i) keep the money in form of deposits at the ECB (with low or even negative rate of return, but risk-free), or ii) invest it in the stock market (whose returns may be more appealing compared to the decreased returns of the securities purchased under the APP), as well as iii) increase the intra-financial interactions via different financial instruments (e.g., increasing intra-financial loan, bond, and equity contracts). This may lead to the alteration of the pattern of intra-financial exposures among financial actors (including banks, investment funds, insurance corporations, pension funds, and other financial institutions), with potential implications in terms of financial stability.

Despite there have been some efforts in the recent macroeconometric literature to investigate the effects of unconventional monetary measures, the exploration of the transmission of the recently implemented APP to the real economy and its implications on financial stability from a complex-network perspective would be of great importance. In particular, a financial network analysis would allow to account for the complex intra-financial interconnections and their potential effects on financial stability, neglected by traditional tools. Network theory has been widely applied in the financial domain to provide useful insights and predictions on the interconnectivity of the financial system and on the propagation of shocks, especially with reference to the banking sector (Eisenberg and Noe 2001; Elsinger et al. 2006; Battiston et al. 2012; Roukny et al. 2013; Battiston et al. 2016). However, less attention has been devoted to the study of the interplay between monetary policy, macroeconomic variables, and network of financial exposures, able to take into account both the interactions between the real and the financial sector and intra-financial interconnectedness (Perillo and Battiston 2017). In particular, in the light of the above context, two fundamental questions deserve more attention in the literature:

1. To what extent, the resources provided to the banking system through QE are transmitted to the real economy?
2. To what extent, the QE may alter the pattern of intra-financial exposures among financial actors and what are the implications in terms of financial stability?

Here, we address these two questions by taking a complex-network perspective to unconventional monetary policy. More specifically, building on (Perillo and Battiston 2017), we first extend the methodology to map the macro-network of financial exposures among the different institutional sectors operating in the euro area (i.e., Eurosystem, Monetary Financial Institutions excluding the Eurosystem, Other Financial Institutions, Investment Funds, Insurance Corporations and Pension Funds, Households, Non-Financial Corporations, and General Governments) and the rest of the world. In particular, differently from Perillo and Battiston (2017), we regard this macro-network as a multilayer weighted network in which the multiple layers correspond to different financial instruments. Second, we illustrate our approach on a novel hand-matched dataset, that we have built based on recently available data by extending our focus to i) loans and deposits, ii) private and public debt securities, iii) APP securities, and iv) shares and other equity. Third, we investigate the financial linkages in the multilayer macro-network of financial exposures both from a static and a dynamic perspective in an attempt to capture i) the transmission of QE to the real economy through the increase of the interactions between the banking sector and the real sector, and ii) the level of intra-financial interconnectedness of the euro area, through the increase of the intra-financial interactions via loans and deposits, securities, and equity, since the implementation of QE. Finally, we extend the assessment of the macroeconomic effects of QE, by exploring the time evolution of i) private and public consumption, ii) private and public investment, iii) Gross Domestic Product (GDP), iv) Harmonised Unemployment Rate (HUR), and v) Harmonised Index of Consumer Prices (HICP). The combination of network variables (i.e., financial linkages of the multilayer macro-network) and macroeconomic variables allows us to shed light on the implications of QE both in terms of stimulation of the real economy and intra-financial interconnectedness. This novel approach represents a tangible step ahead in the comprehensive analysis of the effects of unconventional monetary policy, which until now involved macroeconomic effects or financial markets’ impact, neglecting network effects and their interplay with macroeconomic variables.

The remainder of this paper is organized as follows. In “Methodology” section, we introduce the methodology that we developed to map the multilayer macro-network of financial exposures among the different institutional sectors operating in the euro area and the rest of the world, across the main classes of financial instruments. In “Data” section, we illustrate the employed network and macroeconomic variables. In “Exploratory results” section, we present the exploratory results both from a static and a dynamic perspective. Finally, “Conclusion” section concludes the paper.

## Methodology

In line with the standards of the European System of National and Regional Accounts, we consider the euro area economy as an open economy composed by four macro sectors: i) the financial sector; ii) the non-financial private sector (or the real sector); iii) the non-financial public sector; and iv) the rest of the world. The financial sector comprises i) the *Eurosystem*, i.e., the European Central Bank and the National Central Banks *(ECB&NCB)* of the nineteen countries that have adopted the euro in stage three of Economic and Monetary Union (EMU)^8^; ii) *Monetary Financial Institutions excluding the Eurosystem (MFI excl. ECB&NCB)*, i.e., the private banking system and money market funds; iii) *Other Financial Institutions (OFI)*, such as saving and loan associations^9^, credit unions^10^, shadow banks^11^, Islamic banks^12^, etc.; iv) *Investment Funds other than the Money Market Funds (Non-MMF IF)*; and v) *Insurance Corporations and Pension Funds (IC&PF)*. The real sector includes i) *Households* and non-profit institutions serving households, such as charities and trade unions *(HH)*; and ii) *Non-Financial Corporations (NFC)*. Lastly, the non-financial public sector consists of *General Governments (GG)*, while the sector *Rest of the World (RW)* includes the financial sector, the real sector and the non-financial public sector, at an aggregate level, operating in non-euro area countries, with an outstanding exposure to the institutional sectors operating in the euro area countries. The institutional sectors considered in our study with relative acronyms and definitions are summarized in Additional file 1: Table SI1.

**Fig. 1:**
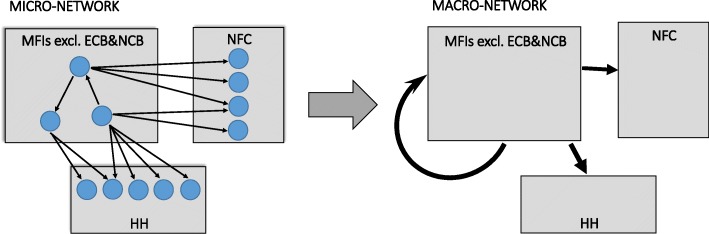
The euro area macro-network of financial exposures as a result of a two-dimension aggegation: by country and by institutional sector. The euro area macro-network of financial exposures is obtained by aggregating i) the nineteen macro-networks of financial exposures among the institutional sectors operating in the nineteen countries belonging to the Eurozone and their mutual interactions, illustrated as shadows of the macro-nodes (i.e., the grey rectangles) in the left-hand side of the picture, and ii) the micro-based granular financial exposures among the different institutions (i.e., the micro-nodes, represented as blue circles) operating within every institutional sector, as represented in the left-hand side of the picture. This two-dimension aggregation leads to the euro area macro-network of financial exposures, represented in the right-hand side of the picture

Building on Perillo and Battiston (2017), we regard the euro area economy described above as a macro-network of financial exposures, where nodes are the aforementioned institutional sectors operating in the euro area (i.e., *Eurosystem, Monetary Financial Institutions excluding the Eurosystem, Other Financial Institutions, Non-MMF Investment Funds, Insurance Corporations and Pension Funds, Households, Non-Financial Corporations, and General Governments*) and the *Rest of the World*. It is worth mentioning that every node is the result of an aggregation on two dimensions: by country and by institutional sector. In particular, the euro area macro-network of financial exposures includes the nineteen macro-networks of financial exposures among the institutional sectors operating in the nineteen countries belonging to the Eurozone and their mutual interactions. Further, every node of the macro-network should be regarded as a network of micro-based granular financial exposures among the different institutions operating within every institutional sector (Perillo and Battiston 2017). Figure 1 visually illustrates the aforementioned process of aggregation.

Differently from Perillo and Battiston (2017), we regard the euro area macro-network of financial exposures as a multilayer weighted network in which the multiple layers correspond to different financial instruments. In particular, our study focuses on four categories of financial instruments: i) loans and deposits; ii) private and public debt securities; iii) APP securities (i.e., private and public securities massively purchased by the Eurosystem from the banking system for monetary policy purposes within the APP 13), and iv) shares and other equity^14^. Links are the financial dependencies through which the different institutional sectors are tied to each other. More specifically, links start from the institutional sector that holds the financial instrument (source) and point to the sector that issued it (target). Weights correspond to our estimate of the direct financial exposures existing among the different institutional sectors through the different financial instruments, based on the balance sheet amount at the end of the period (stock) and their weight on the total financial assets of the holding institutional sector. Formally, direct exposures among financial actors are estimated in the following way:

1 $$ {DE}_{i}= \left(\sum_{j=1}^{n} \beta_{ij}^{Loans\&deposits} + \beta_{ij}^{Securities} + \beta_{ij}^{APP \ securities} + \beta_{ij}^{Equity} \right) + R_{i},  $$

where *β*_*ij*_ denotes the monetary value of the exposure of institutional sector *i* to the institutional sectors *j* via the different considered financial instruments, *n* is the number of institutional sectors (or nodes) considered in our analysis, while *R*_*i*_ is a residual, accounting for the exposures to sectors or via instruments that we are not considering in our analysis.

**Fig. 2:**
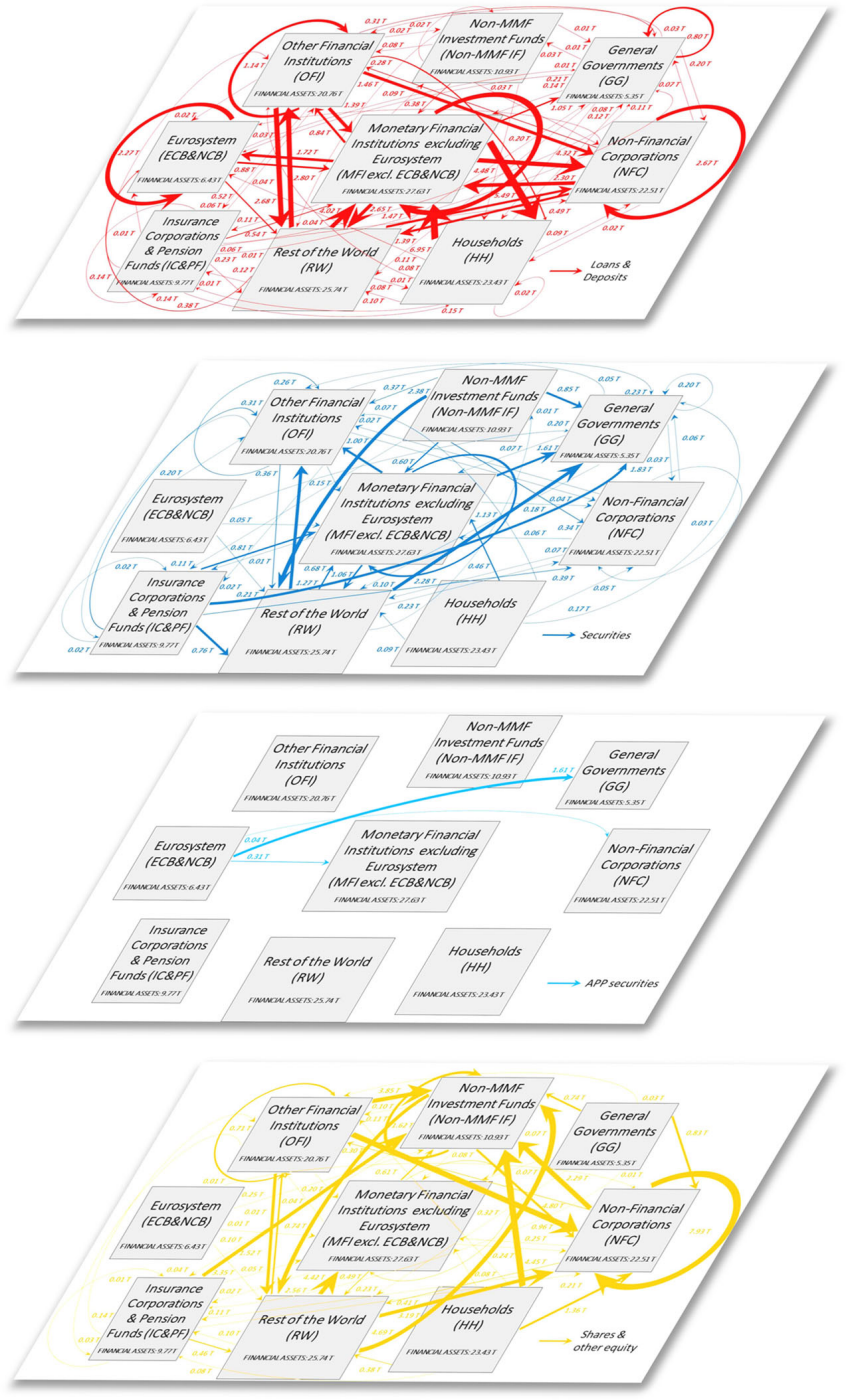
The multilayer macro-network of financial exposures in the euro area; June 2017. Multilayer macro-network of financial exposures among the different institutional sectors operating in the euro area (i.e., Eurosystem (ECB&NCB), Monetary Financial Institutions excluding the Eurosystem (MFI excl. ECB&NCB), Other Financial Institutions (OFI), Non-MMF Investment Funds (Non-MMF IF), Insurance Corporations and Pension Funds (IC&PF), Households (HH), Non-Financial Corporations (NFC), General Governments (GG)), and the Rest of the World (RW), via i) loans and deposits (first layer, red links), ii) private and public debt securities (second layer, blue links), iii) APP securities (third layer, light blue links), and iv) shares and other equity (fourth layer, yellow links), with reference to the second quarter of 2017. Weights represent the outstanding financial exposures of a source institutional sector to a target institutional sector expressed in trillion euros. Data sources: European Central Bank (2017b) and European Central Bank (2017a)

Figure 2 illustrates the multilayer macro-network of financial exposures among the different institutional sectors of the euro area and the rest of the world, via loans and deposits, private and public debt securities, APP securities, and shares and other equity, with reference to the second quarter of 2017.

The analysis of the euro area financial system as a multilayer financial network allows us to obtain relevant insights on the challenging problem of estimating the financial interdependencies existing between the banking sector and both the real and the financial sector. In fact, this approach enables to account for the relevant direct and indirect exposures via the different financial instruments considered together. Further, the multilayer macro-network analysis also provides us with a map of the potential channels through which distress may propagate as a consequence of shocks of different nature. These insights could not be obtained with a single-layer analysis, in fact, by looking at a single layer at a time, one may fail to detect instability, underestimating the systemic importance and the vulnerability of the nodes in the network.

In an attempt to demonstrate that a single-layer analysis may lead to the underestimation of the systemic importance and the vulnerability of the nodes in the network, we compute different network statistics both on the single-layer macro-networks and on the multilayer macro-network of financial exposures. In particular, building on Opsahl et al. (2010), we compute a generalization of the weighted and non-weighted version of the degree, closeness and betweenness centrality measures. These centrality measures are able to take into account both the number of ties, as typical for non-weighted network measures, and the tie weights, as common in weighted network metrics, allowing for attributing different relative importance to the number of ties compared to the tie weighs in contributing to the centrality of a node. More specifically, with reference to the degree centrality, (Opsahl et al. 2010) proposed the following measures as a compromise betweeen the weighted and non-weighted degree metrics:

2 $$ {C}^{w\alpha}_{D-out}(i)= {\left({k}^{out}_{i}\right)}^{(1 - \alpha)} \times {\left({s}^{out}_{i}\right)}^{\alpha}  $$

and

3 $$ {C}^{w\alpha}_{D-in}(i)= {\left({k}^{in}_{i}\right)}^{(1 - \alpha)} \times {\left({s}^{in}_{i}\right)}^{\alpha},  $$

where ${{k}^{out}_{i}}$ and ${{k}^{in}_{i}}$ correspond to the out-degree and in-degree centrality measures, firstly introduced by Freeman (1978), which are generally computed on non-weighted networks, as they are defined as the number of links that originate from node *i*, and the number of links directed to node *i*, respectively. Further, ${{s}^{out}_{i}}$ and ${{s}^{in}_{i}}$ correspond to the out-strenght and in-strenght centrality measures, firstly introduced by Barrat et al. (2004), which are computed on weighted networks, as they are defined as the sum of the weights attached to the outgoing links and to the incoming links of a generic node *i*, respectively. Lastly, *α* is a tuning parameter that allows for attributing different relative importance to the number of ties compared to the tie weighs in contributing to the centrality of a node. For example, when *α*=0, ${C}^{w\alpha }_{D-out}(i)$ and ${C}^{w\alpha }_{D-in}(i)$ values equal the node out- and in-degree $\left (\text {i.e.},\ {{k}^{out}_{i}}\ \text {and}\ {{k}^{in}_{i}}\right)$, while when *α*=1, the ${C}^{w\alpha }_{D-out}(i)$ and ${C}^{w\alpha }_{D-in}(i)$ values equal the node out- and in-strength $\left (\text {i.e.},\ {{s}^{out}_{i}}\ \text {and}\ {{s}^{in}_{i}}\right)$. Therefore, setting *α*=0.5 implies that the same importance is attributed to number of ties and tie weights in contributing to the centrality of a node *i*. Similarly to the degree centrality, (Opsahl et al. 2010) proposed the following formulations for the closeness and the betweenness centrality measures, respectively:

4 $$ {C}^{w\alpha}_{C}(i)= \left(\sum_{j=1}^{n} d^{w \alpha}(i,j) \right)^{-1},  $$

where *d*^*wα*^(*i*,*j*) is shortest path between two nodes *i* and *j* defined as:

5 $$ {d}^{w\alpha}(i,j)= min \left(\frac{1}{\left(w_{ih}\right)^{\alpha}} + \dots + \frac{1}{\left(w_{hj}\right)^{\alpha}} \right),  $$

and

6 $$ {C}^{w\alpha}_{B}(i)= \frac{g^{w \alpha}_{jk}(i)}{g^{w \alpha}_{jk}},  $$

where $g^{w \alpha }_{jk}$ is the number of shortest paths between two nodes (*d*^*wα*^(*j*,*k*)), $g^{w \alpha }_{jk}(i)$ is the number of those paths that go through node *i*, and *α* is the aforementioned tuning parameter.

Further, building on Kaushik and Battiston (2013) and Junker and Schreiber (2011), we compute the weighted version of the Katz centrality, able to measure the influence of nodes taking into account the total number of walks between a pair of nodes, given by:

7 $$ {C}^{w}_{K}(i)= \sum_{k=1}^{\infty} \sum_{j=1}^{n} \gamma^{k} \left(A^{k}\right)_{ij},  $$

where *A* is the weighted adjacency matrix of the network under consideration, the power of *A* indicates the presence (or absence) of links between two nodes through intermediaries, and *γ* is an attenuation factor^15^, employed to penalize connections made with distant neighbors.

Lastly, building on Bardoscia et al. (2015), we estimate a measure of nodes’ vulnerability coming from the generalized version of the DebtRank algorithm, i.e., a measure of systemic impact inspired by feedback centrality, that takes recursively into account the impact of the distress of an initial node across the whole network, proposed in its original formulation by Battiston et al. (2012). The generalized DebtRank financial contagion model was proposed by Bardoscia et al. (2015) as an extension of the original algorithm to allow distress to be propagated along the paths in the network, including all cycles^16^. The financial distress propagation process proposed by Bardoscia et al. (2015) is defined as follows:

8 $$ {h}_{i}(t+1)= min \left\{1, h_{i}(t) + \sum_{j=1}^{n} (1-RR)l^{b}_{ij}\left[h_{j}(t) - h_{j}(t-1)\right]\right\},  $$

where *h*_*i*_(*t*+1) and *h*_*i*_(*t*) correspond to the individual vulnerability of node *i* at time *t*+1 and at time *t*, respectively, *RR* is the recovery rate (i.e., the amount recovered through foreclosure or bankruptcy procedures in the event of a default, expressed as a percentage of face value), $l^{b}_{ij}$ is the leverage ratio of node *i* with respect to *j*, given by the ratio between the assets of node *i* invested in node *j* and the equity (i.e., difference between assets and liabilities) of node *i*, and *h*_*j*_(*t*) and *h*_*j*_(*t*−1) correspond to the vulnerability of node *j* at time *t* and *t*−1, respectively.

## Data

The empirical analysis has been conducted both from a static and a dynamic perspective, with regard to the second quarter of 2017, and the period spanning from the first quarter of 1999 to the second quarter of 2017 (1999Q1-2017Q2, both quarters included), respectively, considering eight institutional sectors operating in the euro area (i.e., *Eurosystem, Monetary Financial Institutions excluding the Eurosystem, Non-MMF Investment Funds, Insurance Corporations and Pension Funds, Households, Non-Financial Corporations, and General Governments*) and the *Rest of the World*^17^. In particular, we have analyzed balance sheet and macroeconomic data from various sources^18^, relative to the nineteen European Union’s Member States belonging to the EMU. Our analysis is based on two categories of variables: i) network variables, i.e., the financial exposures via loan, bond, and equity contracts, through which the euro area sectors are tied to each other; and ii) macroeconomic variables, i.e., i) private and public consumption, ii) private and public investment, iii) Gross Domestic Product (GDP), iv) Harmonised Unemployment Rate (HUR), and v) Harmonised Index of Consumer Prices (HICP). The combination of the network variables and macroeconomic variables allows us to shed light on the implications of QE both in terms of stimulation of the real economy and financial stability. A detailed illustration and definition of the analysed variables, the way how we have computed them and the sources and limitations of row data are provided in the Additional file 1 (Section SI3).

## Exploratory results

### Overview of the euro area economy

**Fig. 3:**
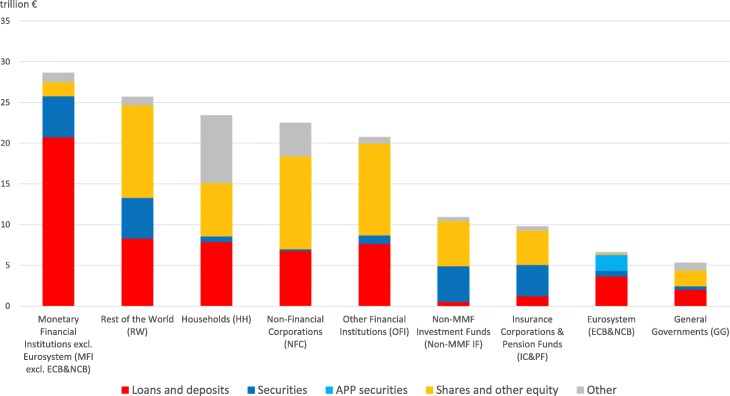
Breakdown of the euro area institutional sectors’ and rest of the world’s financial assets by instrument type; June 2017. Breakdown of the euro area institutional sectors’ financial assets by instrument type, i.e., short-term and long-term loans and deposits (in red), short-term and long-term private and public debt securities (in blue), APP securities (in light blue), listed, unlisted, and investment fund shares and other equity (in yellow), and other financial assets (in grey). The residual category “other” includes i) monetary gold and Special Drawing Rights (SDRs) (totally held by Monetary Financial Institutions), ii) insurance and pension schemes (mostly, i.e., the 93.86%, held by Households), and iii) other accounts receivable and financial derivatives (mostly, i.e., the 50.66%, held by Non-Financial Corporations). Data source: (European Central Bank 2017b)

**Fig. 4:**
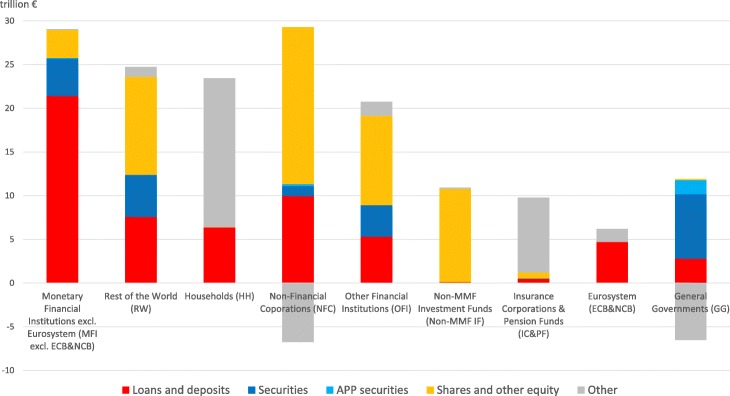
Breakdown of the euro area institutional sectors’ and rest of the world’s financial liabilities by instrument type; June 2017. Breakdown of the euro area institutional sectors’ and rest of the world’s financial liabilities by instrument type, i.e., short-term and long-term loans and deposits (in red), short-term and long-term private and public debt securities (in blue), APP securities (in light blue), listed, unlisted, and investment fund shares and other equity (in yellow), and other financial assets (in grey). The residual category “other” includes i) insurance and pension schemes (mostly, i.e., the 93.78%, issued by Insurance Corporations and Pension Funds), ii) other accounts receivable and financial derivatives (mainly, i.e., the 49.46%, issued by Non-Financial Corporations), and iii) euro area institutional sectors’ net worth. Data source: (European Central Bank 2017b)

Our analysis focuses on the interactions among the different institutional sectors operating in the euro area and the rest of the world via the four main categories of financial instruments: i) short-term and long-term loans and deposits, ii) short-term and long-term private and public debt securities (excluding APP securities), iii) APP securities, and iv) listed, unlisted, and investment fund shares and other equity. For the sake of accuracy, it is worth mentioning that, in order to have a complete overview of total euro area financial assets, we should also take into account the residual category “all remaining assets”. The latter category includes i) monetary gold and Special Drawing Rights (SDRs), ii) insurance and pension schemes, and iii) other accounts receivable and financial derivatives^19^. Figures 3 and 4 illustrate the breakdown of the euro area institutional sectors’ and the rest of the world’s financial assets and liabilities, respectively, by instrument type. As it can be seen from the Figs. 3 and 4, on the one hand, loans and deposits owed in the euro area are mostly granted by *Monetary Financial Institutions* and *Rest of the World*. On the other hand, loans and deposits granted in the euro area are mostly owed by *Monetary Financial Institutions* and *Non-Financial Corporations*. As regards debt securities held and issued in the euro area, while *Monetary Financial Institutions, Rest of the World* and *Non-MMF Investment Funds* are the main holders, *General Governments* and *Rest of the World* are the main issuers. With particular reference to the private and public securities purchased within the APP, not surprisingly, the only holder is the *Eurosystem*, while the main issuer is the institutional sector *General Governments*. In particular, until December 2017, within the APP, the Eurosystem carried out public sector security purchases amounting to about 1.89 trillion euros, out of the total asset purchases amounting to about 2.29 trillion euros (European Central Bank 2017a). The main players of the euro area equity market are *Rest of the World, Non-Financial Corporations*, and *Other Financial Institutions* on the asset side (holdings), and *Non-Financial Corporations, Rest of the World, Non-MMF Investment Funds,* and *Other Financial Institutions* on the liability side (issuances). Lastly, concerning the residual category “other” of the liability side, it includes also the euro area institutional sectors’ net worth. In this regard, it is worth mentioning that it is positive for all the institutional sectors, except *Non-Financial Corporations, General Governments* (significantly negative) and *Non-MMF Investment Funds* (slightly negative), with the highest net worth for *Households* and the lowest for *Non-Financial Corporations*.

### The euro area multilayer macro-network of financial exposures

#### The static perspective

**Fig. 5:**
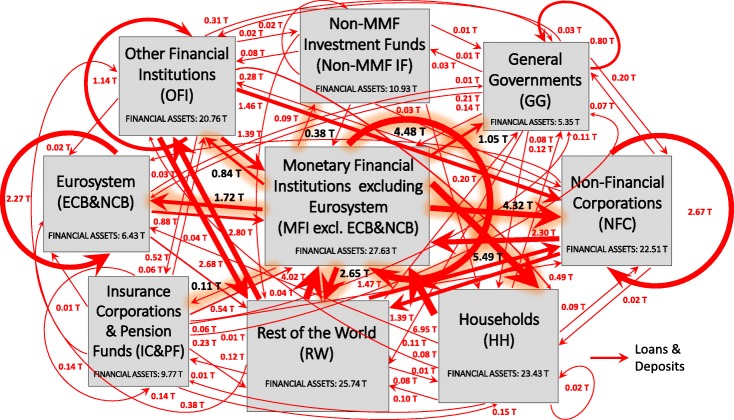
The euro area macro-network of financial exposures via loans and deposits; June 2017. The macro-network of financial exposures among the different institutional sectors operating in the euro area (i.e., Eurosystem (ECB&NCB), Monetary Financial Institutions excluding the Eurosystem (MFI excl. ECB&NCB), Other Financial Institutions (OFI), Non-MMF Investment Funds (Non-MMF IF), Insurance Corporations and Pension Funds (IC&PF), Households (HH), Non-Financial Corporations (NFC), and General Governments (GG)), and the Rest of the World (RW), via short-term and long-term loans and deposits (represented with red links), with reference to the second quarter of 2017. Weights represent the outstanding financial exposures of a source institutional sector to a target institutional sector expressed in trillion euros. Data source: (European Central Bank 2017b)

**Fig. 6:**
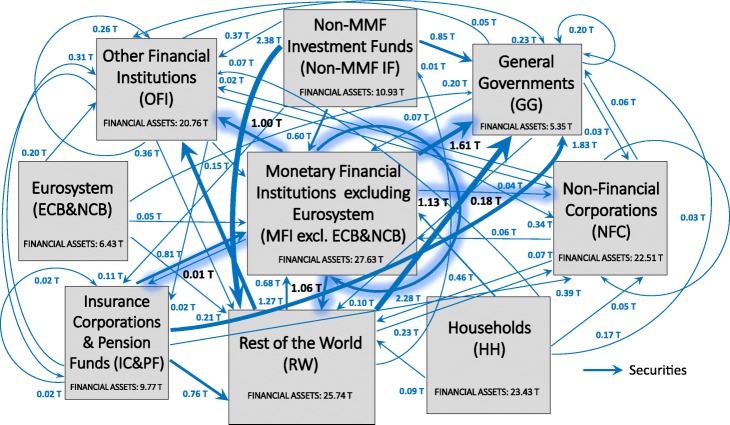
The euro area macro-network of financial exposures via debt securities; June 2017. The macro-network of financial exposures among the different institutional sectors operating in the euro area (i.e., Eurosystem (ECB&NCB), Monetary Financial Institutions excluding the Eurosystem (MFI excl. ECB&NCB), Other Financial Institutions (OFI), Non-MMF Investment Funds (Non-MMF IF), Insurance Corporations and Pension Funds (IC&PF), Households (HH), Non-Financial Corporations (NFC), and General Governments (GG)), and the Rest of the World (RW), via short-term and long-term private and public debt securities (represented with blue links) excluding APP securities, with reference to the second quarter of 2017. Weights represent the outstanding financial exposures of a source institutional sector to a target institutional sector expressed in trillion euros. Data source: (European Central Bank 2017b)

**Fig. 7:**
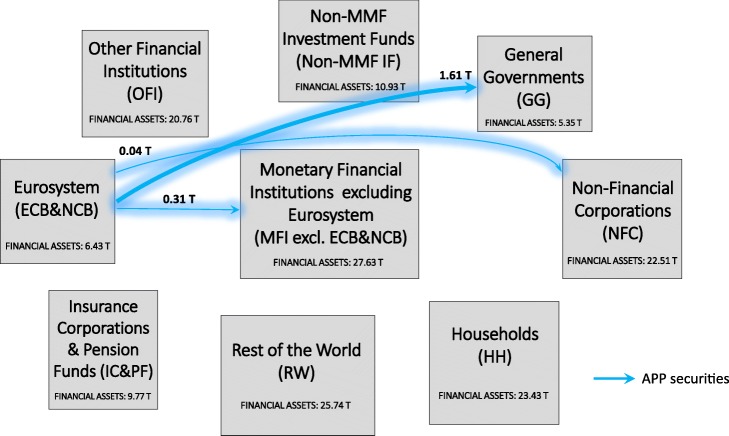
The euro area macro-network of financial exposures via APP securities; June 2017. The macro-network of financial exposures among the different institutional sectors operating in the euro area (i.e., Eurosystem (ECB&NCB), Monetary Financial Institutions excluding the Eurosystem (MFI excl. ECB&NCB), Other Financial Institutions (OFI), Non-MMF Investment Funds (Non-MMF IF), Insurance Corporations and Pension Funds (IC&PF), Households (HH), Non-Financial Corporations (NFC), and General Governments (GG)), and the Rest of the World (RW), via private and public debt securities purchased within the APP (represented with light blue links), with reference to the second quarter of 2017. Weights represent the outstanding financial exposures of a source institutional sector to a target institutional sector expressed in trillion euros. Data source: (European Central Bank 2017b)

**Fig. 8:**
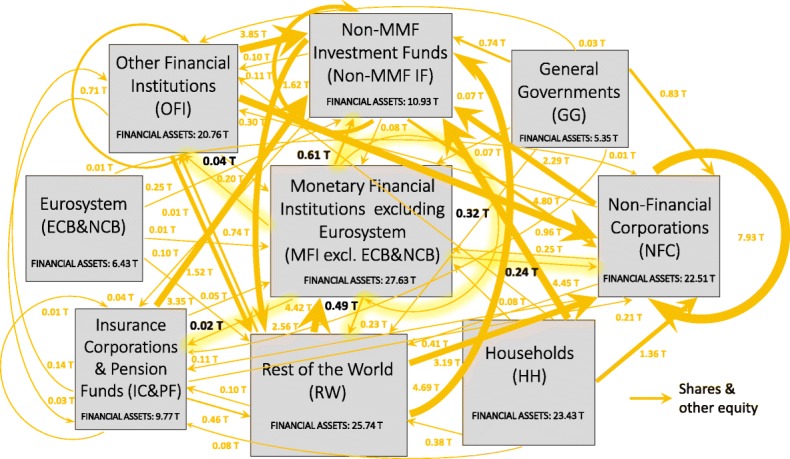
The euro area macro-network of financial exposures via shares and other equity; June 2017. The macro-network of financial exposures among the different institutional sectors operating in the euro area (i.e., Eurosystem (ECB&NCB), Monetary Financial Institutions excluding the Eurosystem (MFI excl. ECB&NCB), Other Financial Institutions (OFI), Non-MMF Investment Funds (Non-MMF IF), Insurance Corporations and Pension Funds (IC&PF), Households (HH), Non-Financial Corporations (NFC), and General Governments (GG)), and Rest of the World (RW), via listed, unlisted, and investment fund shares and other equity (represented with yellow links), with reference to the second quarter of 2017. Weights represent the outstanding financial exposures of a source institutional sector to a target institutional sector expressed in trillion euros. Data source: (European Central Bank 2017b)

**Fig. 9:**
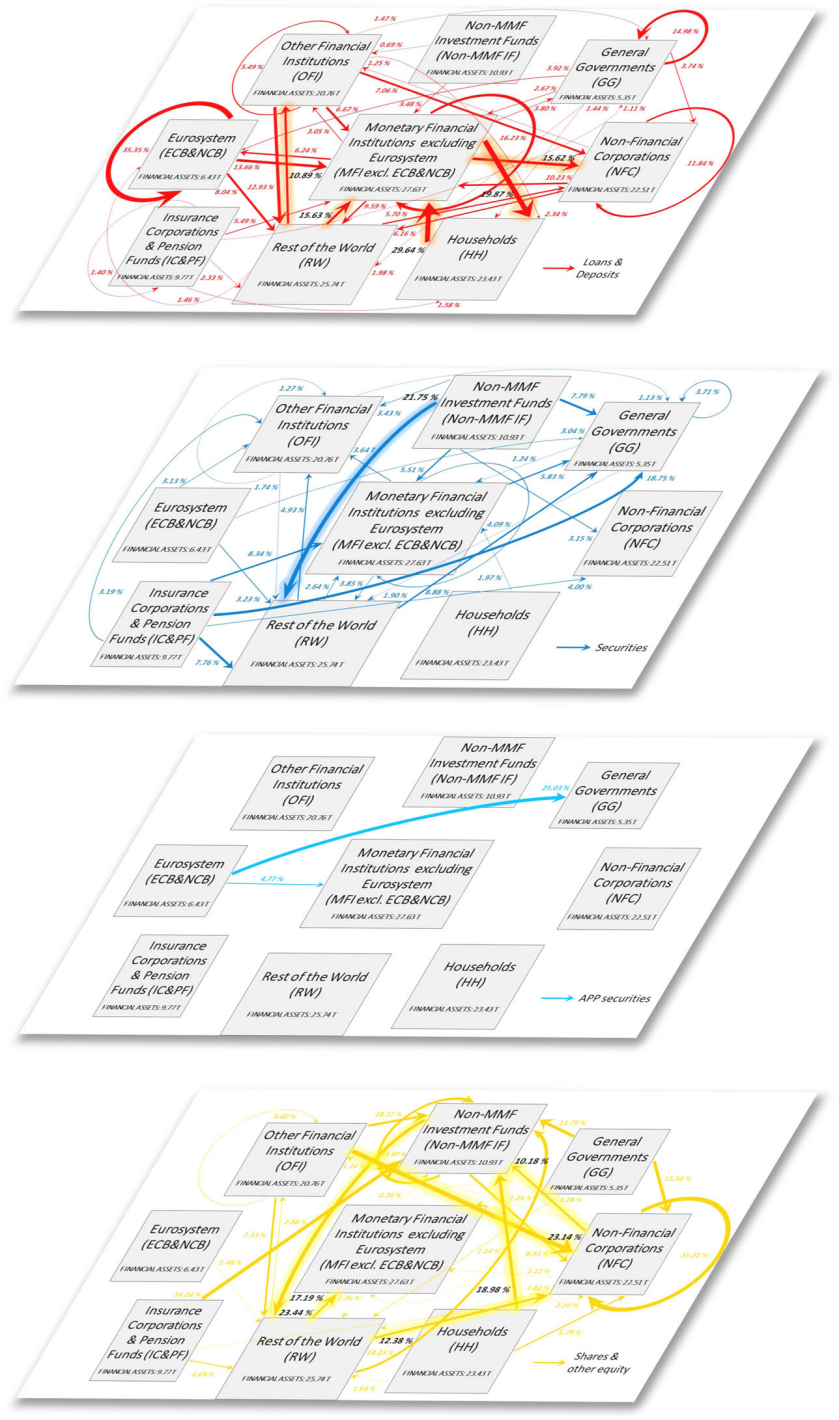
The multilayer macro-network of financial exposures in the euro area; June 2017. Multilayer macro-network of financial exposures among the different institutional sectors operating in the euro area (i.e., Eurosystem (ECB&NCB), Monetary Financial Institutions excluding the Eurosystem (MFI excl. ECB&NCB), Other Financial Institutions (OFI), Non-MMF Investment Funds (Non-MMF IF), Insurance Corporations and Pension Funds (IC&PF), Households (HH), Non-Financial Corporations (NFC), General Governments (GG)), and the Rest of the World (RW), via i) loans and deposits (first layer, red links), ii) private and public debt securities (second layer, blue links), iii) APP securities (third layer, light blue links), and iv) shares and other equity (fourth layer, yellow links), with reference to the second quarter of 2017. Weights represent the outstanding financial exposures of a source institutional sector to a target institutional sector and they are normalized by total assets of the source. Data sources: (European Central Bank 2017b) and (European Central Bank 2017a)

In an attempt to investigate the implications of QE both in terms of stimulation of the real economy and intra-financial interconnectedness, we first analyse the euro area multilayer macro-network of financial exposures from a static perspective. In particular, first, we separately investigate the different layers of the euro area multilayer macro-network of financial exposures, with a particular focus on direct exposures. Second, we focus on the comprehensive analysis of the euro area financial system as a multilayer macro-network, by considering direct and indirect financial exposures among institutional sectors across the different financial instruments. Figures 5, 6, 7 and 8 illustrate the macro-networks of financial exposures among the different institutional sectors of the euro area and the rest of the world, with reference to the second quarter of 2017, via loans and deposits, debt securities, APP securities, and shares and other equity, respectively, while Fig. 9 illustrates the euro area as a multilayer macro-network of financial exposures via all the aforementioned financial instruments. Links are the financial dependencies through which the different institutional sectors are tied to each other. In particular, links start from the institutional sector that holds the financial instrument and point to the sector that issued it. Weights correspond to the balance sheet outstanding amount at the end of the period (stocks) of loan and deposit, debt security, APP security, and share holdings/issuances, and they are expressed in trillion euros in Figs. 5, 6, 7 and 8, and as a percentage of the total assets of the source institutional sector in Fig. 9. Due to the fact that our main aim is to investigate whether the resources received by banks through QE are actually stimulating the real economy via loans, bonds, and equity or they are staying into the financial system, our main focus is on the links starting from the node *Monetary Financial Institutions excluding the Eurosystem*.

As shown in Fig. 5, at the end of June 2017, the largest fraction of loans and deposits granted by *Monetary Financial Institutions excluding the Eurosystem* were loans to *Households*, followed by bank-to-bank loans and deposits and loans to *Non-Financial Corporations*, amounting to around 5.49, 4.48, and 4.32 trillion euros, respectively. More specifically, from the macro-network analysis of the financial exposures via loans and deposits, we can notice that, despite the fact that a large fraction of loans and deposits granted by *Monetary and Financial Institutions* is intra-financial (7.53 trillion euros), the most relevant portion is granted by the private banking sector to the real sector (9.81 trillion euros), followed by loans and deposits to the *Rest of the World* and public sector (amounting to 2.65 and 1.05 trillion euros, respectively). Unfortunately, available data on the institutional sector *Rest of the World* do not allow us to discriminate between real sector and financial sector of non-euro area countries receiving the aforementioned amount of loans and deposits by the euro area private banking system. Moreover, it is worth mentioning that, the disaggregation of Monetary Financial Institutions in *Eurosystem* and *Monetary Financial Institutions excluding the Eurosystem*, not only from the point of view of the sector granting the loans and deposits, such as in Perillo and Battiston (2017), but also from the perspective of the institutional sector receiving them, allows us to discriminate among bank-to-bank loans and deposits (4.48 trillion euros), loans and deposits granted by the private banking system to the Eurosystem (1.72 trillion euros), and loans and deposits granted and received within the Eurosystem (2.27 trillion euros). The latter category belongs to the so-called TARGET balances, i.e., intra-Eurosystem assets and liabilities of central banks’ balance sheet resulting from net cross-border payments in the form of central bank reserves, via the Eurosystem real-time payment system, which settles continuously euro-denominated payments on an individual transaction-by-transaction basis without netting (called TARGET2)(Eisenschmidt et al. 2017).

Table 1 reports some network statistics (see “Methodology” section) computed on the loan and deposit macro-network of financial exposures. In particular, we have computed the generalized version of the degree, closeness and betweenness centrality measures defined in Eqs. 2–6, after setting the tuning parameter *α* equal to 0.5. Further, we have calculated the Katz centrality given by Eq. 7, and a measure of nodes’ vulnerability coming from the generalized version of DebtRank defined by Eq. 8, where we have set the initial shock equal to 0.1, and the recovery rate equal to its conventional value of 0.6. Moreover, due to the fact that three of the institutional sectors under consideration exhibit a negative equity, we have estimated the financial distress propagation process (Eq. 8) by replacing the leverage matrix with the matrix of the financial exposures via which the different institutional sectors are tied to each other relative to the total financial assets of the source institutional sector (Castrén and Rancan 2014). As shown in Table 1, *Monetary and Financial Institutions excluding the Eurosystem* exhibit the highest out-degree and in-degree. This result is confirmed by the closeness, betweenness, and Katz centrality measures, according to which the most central node in the loan and deposit macro-network is *Monetary and Financial Institutions excluding the Eurosystem*. Moreover, from the computation of the DebtRank financial distress propagation process, we can derive that *Monetary and Financial Institutions excluding the Eurosystem* is also the most vulnerable node in the loan and deposit macro-network.

**Table 1:** Network statistics - loan and deposit layer

Institutional sectors	${C}^{w\alpha }_{D-out}$	${C}^{w\alpha }_{D-in}$	${C}^{w\alpha }_{C}$	${C}^{w\alpha }_{B}$	${C}^{w}_{K}$	*h*_*i*_(*t*+1)
Non-financial corporations	7.80	9.62	0.07	0	907.94	0.14
Eurosystem	5.76	6.51	0.06	0	323.65	0.17
Monetary financial institutions excluding the Eurosystem	13.67	13.77	0.09	41	1504.91	0.19
Non-MMF investment funds	2.26	1.04	0.05	0	13.20	0.11
Other financial institutions	8.25	6.85	0.07	0	304.61	0.14
Insurance corporations and pension funds	3.38	2.01	0.05	0	23.31	0.12
General governments	3.78	4.90	0.04	0	184.57	0.14
Households	8.14	7.57	0.08	0	750.54	0.14
Rest of the world	7.97	7.85	0.08	4	558.55	0.14

As regards the security layers, on the one hand, Fig. 6 shows that the largest fraction of securities held by *Monetary Financial Institutions excluding the Eurosystem*, amounting to 1.61 trillion euros, were issued by *General Governments*, followed by debt securities issued by *Monetary Financial Institutions excluding the Eurosystem* (1.13 trillion euros), *Rest of the World* (1.06 trillion euros), and *Other Financial Institutions* (1.00 trillion euros). *Non-financial Corporations* and *Insurance Corporations and Pension Funds* play only a minor role as issuing counterparties of the securities held by *Monetary Financial Institutions excluding the Eurosystem*. The fact that *General Governments* play a key role as an issuing counterparty of the securities held by *Monetary Financial Institutions excluding the Eurosystem* could be explained as follows. The expectation of the public security purchases by the Eurosystem and the subsequent increase in the prices of the purchased securities may have generated an increase in the purchases of public securities by the private banking sector in order to sell them to the *Eurosystem*. However, our data, being data on holdings and issuances, are not able to provide empirical evidence of the aforementioned speculation. On the other hand, with particular reference to the APP securities, as we can see from Fig. 7, at the end of the second quarter of 2017, the largest fraction of APP securities held by the *Eurosystem* was issued by the institutional sector *General Governments*, amounting to 1.61 trillion euros, followed by securities issued by *Monetary Financial Institutions excluding the Eurosystem* (0.31 trillion euros), and *Non-Financial Corporations* (0.04 trillion euros). In fact, the majority of purchases carried out by the Eurosystem within the APP until now have been public sector security purchases (European Central Bank 2017a).

Tables 2 and 3 report some network statistics (see “Methodology” section) computed on the debt security and APP security macro-networks of financial exposures, respectively. On the one hand, as we can see from Table 2, in the debt security macro-network *Monetary Financial Institutions excluding the Eurosystem* exhibit the highest out-degree centrality, while *General Governments* exhibit the largest in-degree centrality. Moreover, from the computation of the closeness centrality, we can derive that the most central nodes in the debt security layer are *Non-MMF Investment Funds and Insurance Corporations and Pension Funds*, while the betweenness and the Katz centrality measures suggest *Rest of the World* and *General Governments*, respectively, as the most central ones. On the other hand, with reference to the APP security macro-network, Table 3 shows that the *Eurosystem* exhibits the largest out-degree and closeness centrality, while *General Governments* exhibit the highest in-degree, which is consistent with the estimate of the Katz centrality measure. Lastly, with reference to the DebtRank financial distress propagation model, while *Non-MMF Investment Funds* and *Insurance Corporations and Pension Funds* are the most vulnerable nodes in the debt security macro-network, the *Eurosystem* exhibits the highest vulnerability in the APP security macro-network.

**Table 2:** Network statistics - debt security layer

Institutional sectors	${C}^{w\alpha }_{D-out}$	${C}^{w\alpha }_{D-in}$	${C}^{w\alpha }_{C}$	${C}^{w\alpha }_{B}$	${C}^{w}_{K}$	*h*_*i*_(*t*+1)
Non-financial corporations	1.22	3.21	0.01	0	2434.44	0.10
Eurosystem	1.81	0	0.02	0	0	0.11
Monetary financial institutions excluding the Eurosystem	5.48	6.01	0.02	6	10074.38	0.12
Non-MMF investment funds	5.23	0.12	0.04	0	60.99	0.14
Other financial institutions	2.52	5.66	0.02	7	12313.89	0.11
Insurance corporations and pension funds	4.97	0.74	0.04	0	192.56	0.14
General governments	1.63	8.18	0.01	0	21109.68	0.11
Households	2.24	0	0.02	0	0	0.10
Rest of the world	4.73	6.34	0.03	17	8617.51	0.12

**Table 3:** Network statistics - APP security layer

Institutional sectors	${C}^{w\alpha }_{D-out}$	${C}^{w\alpha }_{D-in}$	${C}^{w\alpha }_{C}$	${C}^{w\alpha }_{B}$	${C}^{w}_{K}$	*h*_*i*_(*t*+1)
Non-financial corporations	0	0.19	0	0	36916.35	0.10
Eurosystem	2.42	0	0.02	0	0	0.13
Monetary financial institutions excluding the Eurosystem	0	0.55	0	0	306468.65	0.10
Non-MMF investment funds	0	0	0	0	0	0.10
Other financial institutions	0	0	0	0	0	0.10
Insurance corporations and pension funds	0	0	0	0	0	0.10
General governments	0	1.27	0	0	1609327	0.10
Households	0	0	0	0	0	0.10
Rest of the world	0	0	0	0	0	0.10

Concerning the equity layer, represented in Fig. 8, at the end of June 2017, the largest exposure exhibited by *Monetary Financial Institutions excluding the Eurosystem* via shares and other equity was to *Non-MMF Investment Funds*, amounting to 0.61 trillion euros, followed by *Rest of the World* (0.49 trillion euros), *Monetary Financial Institutions excluding the Eurosystem* (0.32 trillion euros), and *Non-Financial Corporations* (0.24 trillion euros). More specifically, from the macro-network analysis of the financial exposures via shares and other equity, we can notice that a very large part of the equity held by the private banking system is intra-financial (0.99 trillion euros), followed by equity issued by the *Rest of the World*^20^ and the real sector (amounting to 0.49 and 0.24 trillion euros, respectively). This result could be interpreted as concerning for the following reason. The larger fraction of bank equity investments in the financial sector compared to the real sector seems to suggest the private banks’ preference for investments in financial services rather than in non-financial goods and services. This is consistent with the process of financialization, according to which, a large fraction of bank resources are addressed to finance (Allahrakha et al. 2015), that mostly funds the purchase of assets that already exist, rather than to businesses, that can employ them in financing new capital investments (Turner 2017).

Table 4 reports some network statistics (see “Methodology” section) computed on the equity macro-network of financial exposures. As shown in Table 4, according to the out-degree and Katz centrality the most central node in the equity macro-network is *Non-Financial Corporations*. However, this result is not confirmed by the in-degree, the closeness, nor the betweenness centrality measures, according to which the most central nodes in the equity layer are *Non-MMF Investment Funds, Other Financial Institutions*, and *Rest of the World*, respectively. Lastly, according to the DebtRank financial distress propagation process, the most vulnerable node in the equity macro-network is *Other Financial Institutions*.

**Table 4:** Network statistics - equity layer

Institutional sectors	${C}^{w\alpha }_{D-out}$	${C}^{w\alpha }_{D-in}$	${C}^{w\alpha }_{C}$	${C}^{w\alpha }_{B}$	${C}^{w}_{K}$	*h*_*i*_(*t*+1)
Non-financial corporations	8.23	13.26	0.05	2	2875.38	0.16
Eurosystem	0.86	0	0.02	0	0	0.10
Monetary financial institutions excluding the Eurosystem	3.21	7.02	0.03	0	363.76	0.11
Non-MMF investment funds	5.67	13.95	0.05	11	1325.79	0.16
Other financial institutions	8.21	4.30	0.07	0	165.18	0.17
Insurance corporations and pension funds	4.96	2.12	0.05	0	47.76	0.15
General governments	3.65	0.02	0.04	0	0	0.14
Households	6.22	0	0.06	0	0	0.13
Rest of the world	8.11	7.01	0.07	18	541.47	0.16

Lastly, Fig. 9 provides us with a comprehensive picture of the existing financial exposures among the different institutional sectors operating in the euro area and the rest of the world across the considered financial instruments. The comprehensive analysis of the euro area financial system as a multilayer macro-network allows us to obtain further insights on the challenging problem of estimating the financial interdependencies existing between the banking sector and both the real and the financial sector, enabling the consideration of the relevant indirect exposures via the different financial instruments considered together. Further, this analysis provides us also with a map of the potential channels through which distress may propagate as a consequence of shocks of different nature. Both the insights on the estimate of financial interdependencies and the map of the potential distress propagation channels could not be obtained with a single layer analysis. In order to make more homogeneous the different layers of the multilayer macro-network of financial exposures, we compute weights as percentages of the total assets of the source institutional sectors. Moreover, for the sake of clarity, we remove links with a weight below 1%. By focusing on the most relevant links (with a weight above 10%) connecting the different institutional sectors of the euro area via loans and deposits, debt securities, and equity, we can identify the existence of relevant indirect exposures of the banking system to the financial sector. In particular, as shown in Fig. 9, *Monetary Financial Institutions excluding the Eurosystem* exhibit a direct exposure amounting to about 16% of their total financial assets to *Non-Financial Corporations*, via loans and deposits. In turn, *Non-Financial Corporations* are exposed for about 10% of their total financial assets to *Non-MMF Investment Fund* equity. The chain continues both via equity and debt securities to the *Rest of the World* arriving i) to *Other Financial Institutions* via loans and to *Non-Financial Corporations* via equity, or ii) coming back to *Monetary Financial Institutions excluding the Eurosystem* via loans and equity, or iii) to *Non-Financial Corporations* via equity, making restart the chain from the beginning in all three cases. Further, *Monetary Financial Institutions excluding the Eurosystem* also exhibit a direct exposure amounting to about the 20% of their total financial assets to *Households*, via loans and deposits. In turn, *Households*, other than being exposed to *Monetary Financial Institutions excluding the Eurosystem* for about 30% of their total financial assets via loans and deposits, are exposed for about 19% of their total financial assets to *Non-MMF Investment Fund* equity. From this point the chain starts to coincide with the previous one. These findings shed light on the existence of indirect exposures of the banking system to the financial sector, and in particular, to *Non-MMF Investment Funds*, and *Other Financial Institutions*, that could not be identified if the financial system was not regarded as a multilayer financial network, leading to an underestimation of the exposures of the banking sector to the financial sector and the potential associated risks.

Table 5 reports some network statistics (see “Methodology” section) computed on the multilayer macro-network of financial exposures via loans and deposits, debt securities, APP securities, and equity, whose matrix of financial exposures has been obtained with Equation 1. As shown in Table 5, according to the out-degree and betweenness centrality measures the most central node in the multilayer macro-network is *Monetary Financial Institutions excluding the Eurosystem*. However, this result is not confirmed by the closeness centrality measure, according to which the most central node in the multilayer macro-network is *Rest of the World*, nor by the in-degree and the Katz centrality measures, according to which the most influential node is *Non-Financial Corporations.* Lastly, from the estimate of the DebtRank financial contagion model, we can derive that the most vulnerable nodes in the multilayer macro-network of financial exposures are *Eurosystem* and *Rest of the World*. Although the aforementioned centrality measures did not always exhibit consistent results among each other, as they are supposed to capture different characteristics of the network structure contributing differently to the concept of centrality, this network analysis allows us to further strengthen the rationale behind the choice of performing a multilayer macro-network analysis. In fact, the differences in the results provided by all the network measures in the single-layer macro-networks and the multilayer macro-network of financial exposures confirm our view, according to which by looking at a single layer at a time, one can fail to detect instability by underestimating the systemic importance of the nodes in the network. Further, the results coming from the estimate of the DebtRank financial contagion model in the single-layer macro-networks and the multilayer macro-network of financial exposures, provide us with empirical evidence on the fact that a single-layer analysis, compared to the multilayer one, may also lead to the underestimation of the vulnerability of the nodes in the network and, therefore, of the potential associated risks.

**Table 5:** Network statistics - multilayer macro-network

Institutional sectors	${C}^{w\alpha }_{D-out}$	${C}^{w\alpha }_{D-in}$	${C}^{w\alpha }_{C}$	${C}^{w\alpha }_{B}$	${C}^{w}_{K}$	*h*_*i*_(*t*+1)
Non-financial corporations	12.83	16.74	0.10	2	1088.06	0.23
Eurosystem	7.60	6.51	0.08	0	98.11	0.26
Monetary financial institutions excluding the Eurosystem	15.73	16.67	0.14	19	785.15	0.25
Non-MMF investment funds	9.72	13.99	0.11	0	447.05	0.25
Other financial institutions	13.36	9.88	0.12	0	285.41	0.25
Insurance corporations and pension funds	9.25	3.02	0.12	0	23.94	0.25
General governments	5.95	10.27	0.07	0	237.42	0.21
Households	11.48	7.57	0.12	1	239.45	0.20
Rest of the world	14.61	12.29	0.16	14	484.75	0.26

#### The dynamic perspective

**Fig. 10:**
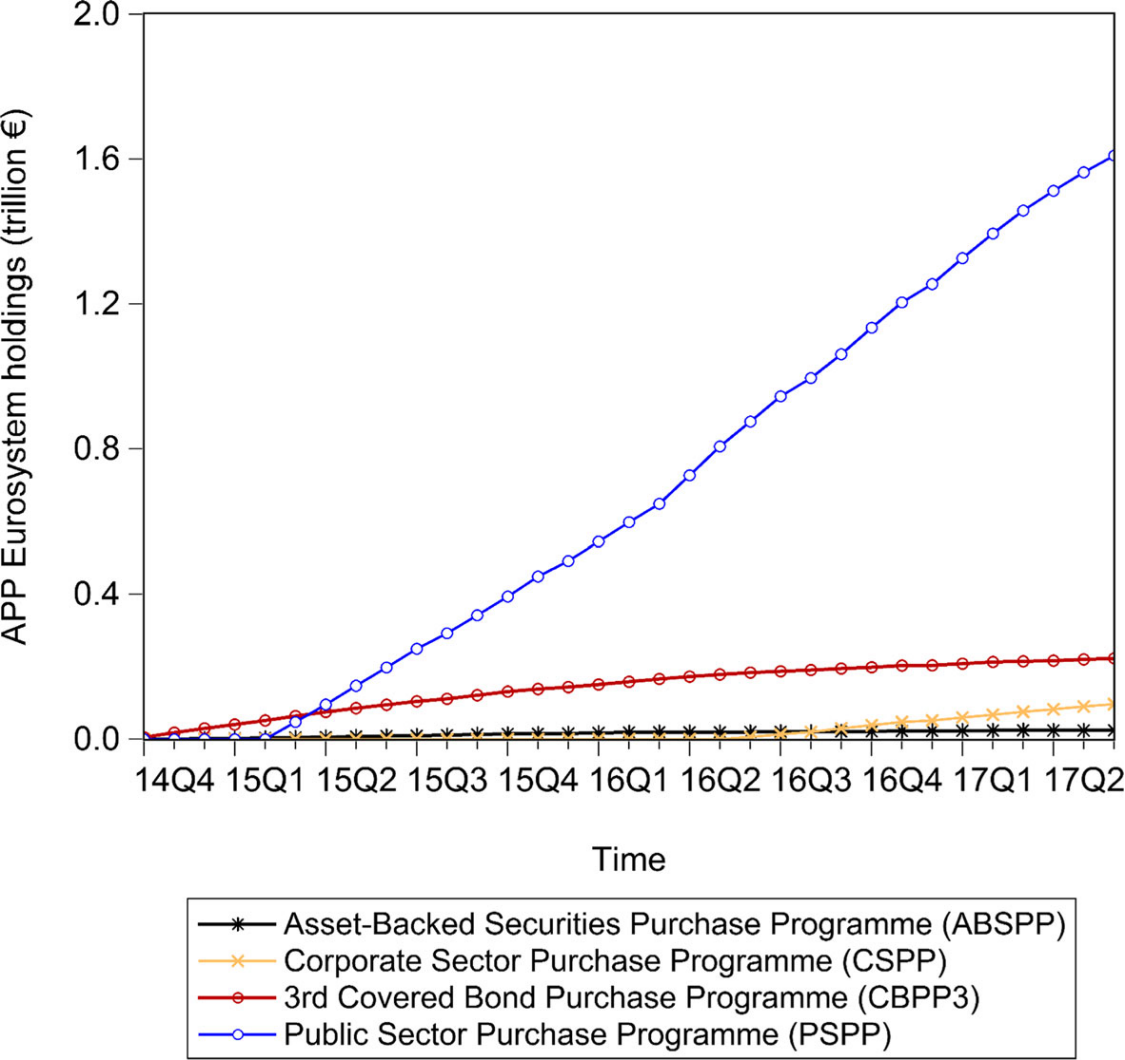
Eurosystem APP holdings - breakdown by purchase programme; 2014Q4-2017Q2. Time series of the breakdown of the Eurosystem APP holdings in the different programmes included in the APP: i) 3rd Covered Bond Purchase Programme (CBPP3, in dark red), ii) Asset-Backed Securities Purchase Programme (ABSPP, in black), iii) Public Sector Purchase Programme (PSPP, in blue), and iv) Corporate Sector Purchase Programme (CSPP, in yellow), over the reference period 2014Q4-2017Q2. Data Source: (European Central Bank 2017a)

**Fig. 11:**
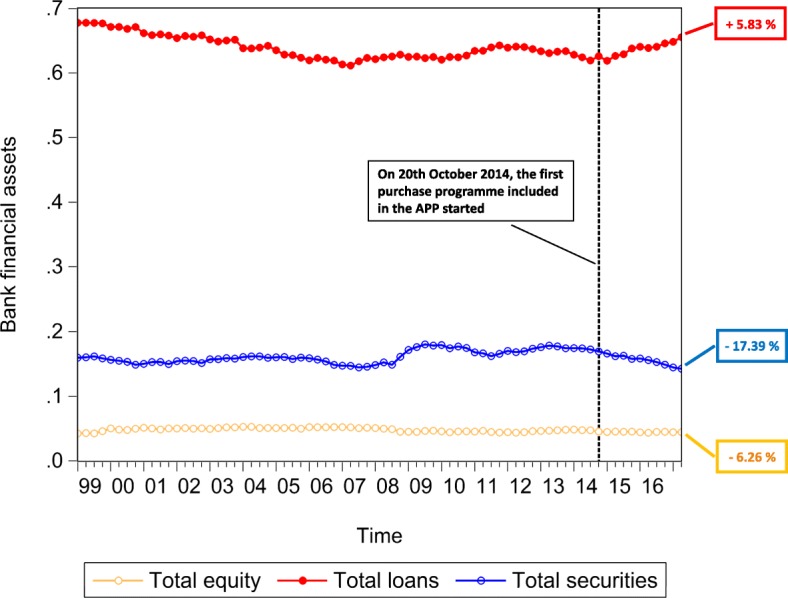
Bank financial assets - breakdown by financial instrument; 1999Q1-2017Q2. Time series of the breakdown of the financial assets held by Monetary Financial Institutions excluding the Eurosystem by financial instrument, i.e., short-term and long-term loans and deposits (in red), short-term and long-term private and public debt securities (in blue), and listed, unlisted, and investment fund shares and other equity (in yellow), over the reference period 1999Q1-2017Q2. The aforementioned time series are normalized by Monetary Financial Institutions excluding the Eurosystem total financial assets. The vertical dashed line corresponds to the beginning of the first purchase programme (3rd Covered Bond Purchase Programme, CBPP3) included in the APP, started in 2014Q4, while the percentages on the right-hand side of the graph correspond to our estimate of the growth rates of the bank holdings of the three categories of financial instruments, since the initiation of QE. Data source: (European Central Bank 2017b)

The multilayer macro-network analysis of the euro area from a static perspective provides us with empirical evidence on the pattern of financial exposures among institutional sectors and across financial instruments with reference to the second quarter of 2017. However, this analysis can only capture the current situation in terms of financial exposures. Therefore, in order to explore the stimulation of the real economy and the evolution of the pattern of intra-financial exposures since the implementation of QE, we investigate the time evolution of the financial linkages in the multilayer macro-network of the euro area. In particular, first, we explore the impact of asset purchases, performed by the Eurosystem from the banking system (whose breakdown in the different programmes included in the APP is represented in Fig. 10), on the private banking system loan and deposit, debt security, and equity holdings, represented in Fig. 11. Second, we investigate the time evolution of the breakdown of loans and deposits granted by *Monetary Financial Institutions excluding the Eurosystem* by receiving counterparty sector, over the reference period 1999Q1-2017Q2, represented in Fig. 11. Third, we explore the time evolution of the breakdown of bank private and public securities, and shares and other equity by issuing counterparty sector, over the reference period 2013Q4-2017Q2, represented in Figs. 13 and 14, respectively. Our main focus is on the fraction of intra-financial loans, securities, and equity, compared to the fraction of financial-real loans, securities, and equity for two main reasons. First, the fraction of financial-real loans, securities, and equity enables us to explore the stimulation of the real economy through the increase of the interactions between the banking sector and the real sector, via loans and deposits, securities and equity, since the implementation of QE. Second, the fraction of intra-financial loans, securities, and equity allows us to investigate the evolution of intra-financial interconnectedness in the euro area, through the increase of intra-financial interactions via loans and deposits, securities and equity, since the implementation of QE.

**Fig. 12:**
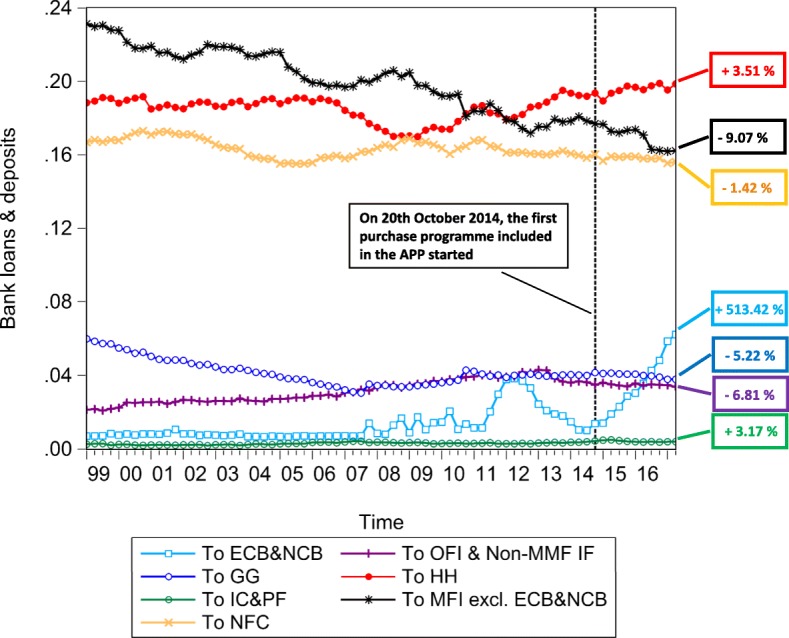
Bank loan and deposit holdings - breakdown by counterparty sector; 1999Q1-2017Q2. Time series of the breakdown of loans and deposits granted by the euro area Monetary Financial Instutions excluding the Eurosystem by counterparty sector, i.e., Eurosystem (ECB&NCB, in light blue), Monetary Financial Institutions excluding the Eurosystem (MFI excl. ECB&NCB, in black), Other Financial Institutions and Non-MMF Investment Funds (OFI & Non-MMF IF, in purple), Insurance Corporations and Pension Funds (IC&PF, in green), Households (HH, in red), Non-Financial Corporations (NFC, in yellow), and General Governments (GG, in blue), over the reference period 1999Q1-2017Q2. The aforementioned time series are normalized by Monetary Financial Institutions excluding the Eurosystem total financial assets. The vertical dashed line corresponds to the beginning of the first purchase programme (3rd Covered Bond Purchase Programme, CBPP3) included in the APP, started in 2014Q4, while the percentages on the right-hand side of the graph correspond to our estimate of the growth rates of bank exposures in terms of loans and deposits to the different institutional sectors of the euro area, since the initiation of QE. Data source: (European Central Bank 2017b)

**Fig. 13:**
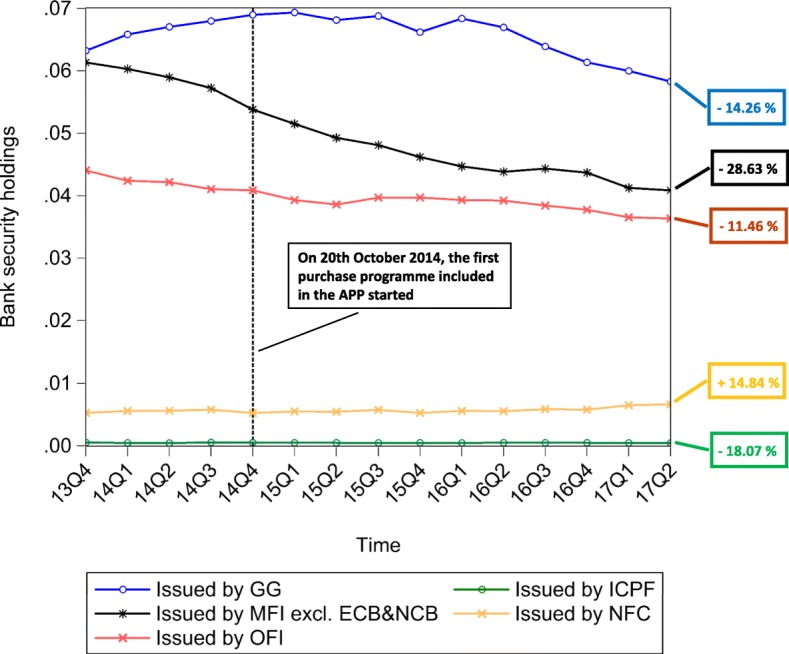
Bank security holdings - breakdown by counterparty sector; 2013Q4-2017Q2. Time series of the breakdown of debt securities (including short-term and long-term private and public securities) held by the euro area Monetary Financial Instutions excluding the Eurosystem by issuing counterparty sector, i.e., Monetary Financial Institutions excluding the Eurosystem (MFI excl. ECB&NCB, in black), Other Financial Institutions (OFI, in pink), Insurance Corporations and Pension Funds (IC&PF, in green), Non-Financial Corporations (NFC, in yellow), and General Governments (GG, in blue), over the reference period 2013Q4-2017Q2. The aforementioned time series are normalized by Monetary Financial Institutions excluding the Eurosystem total financial assets. The vertical dashed line corresponds to the beginning of the first purchase programme (3rd Covered Bond Purchase Programme, CBPP3) included in the APP, started in 2014Q4, while the percentages on the right-hand side of the graph correspond to our estimate of the growth rates of bank exposures in terms of debt securities to the different institutional sectors of the euro area, since the initiation of QE. Data source: (European Central Bank 2017b)

**Fig. 14:**
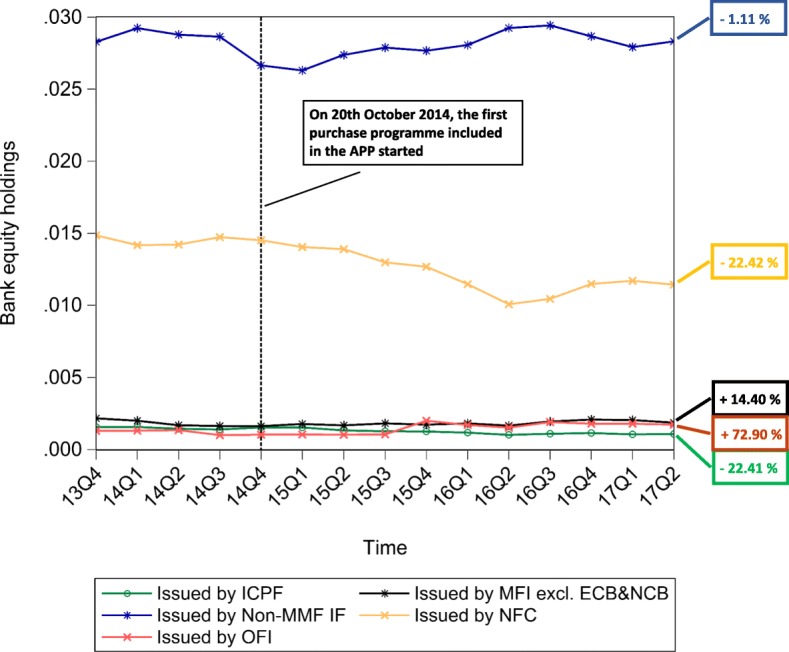
Bank equity holdings - breakdown by counterparty sector; 2013Q4-2017Q2. Time series of the breakdown of equity (including listed, unlisted, and investment fund shares) held by the euro area Monetary Financial Instutions excluding the Eurosystem by issuing counterparty sector, i.e., Monetary Financial Institutions excluding the Eurosystem (MFI excl. ECB&NCB, in black), Other Financial Institutions (OFI, in pink), Non-MMF Investment Funds (Non-MMF IF, in dark blue), Insurance Corporations and Pension Funds (IC&PF, in green), and Non-Financial Corporations (NFC, in yellow), over the reference period 2013Q4-2017Q2. The aforementioned time series are normalized by Monetary Financial Institutions excluding the Eurosystem total financial assets. The vertical dashed line corresponds to the beginning of the first purchase programme (3rd Covered Bond Purchase Programme, CBPP3) included in the APP, started in 2014Q4, while the percentages on the right-hand side of the graph correspond to our estimate of the growth rates of bank exposures in terms of equity to the different institutional sectors of the euro area, since the initiation of QE. Data source: (European Central Bank 2017b)

In Figs. 11, 12, 13 and 14, the time series of bank holdings are normalized by*Monetary Financial Institutions excluding the Eurosystem* total financial assets, the vertical dashed line corresponds to the beginning of the first purchase programme included in the APP, started in the fourth quarter of 2014, and the percentages on the right-hand side of the graphs correspond to our estimate of the growth rates of bankfinancial exposures via the different financial instruments (Fig. 11) to the different euro area institutional sectors (Figs. 12, 13 and 14). In particular, the growth rates have been estimated in the following way:

9 $$ {g}_{t}=\frac{Y_{t}-Y_{t-\tau}}{Y_{t-\tau}} \times 100  $$

where *g*_*t*_ is the growth rate of the variable under consideration at time *t*, *Y*_*t*_ is the value of the variable under consideration at time *t*, and *Y*_*t*−*τ*_ is the value of the same variable at time *t*−*τ*.In our case, *t* corresponds to the second quarter of 2017, while *t*−*τ* corresponds to the starting point of QE, that we have identified with the fourth quarter 2014, due to the fact that the first purchase programme included in the APP started on 20th October 2014. As we can see from Fig. 11, since QE, while bank lending activity(represented in red) has increased by the 5.83%, bank financial exposures via debt securities (represented in blue) and equity (represented in yellow) to the euro area economy have decreased by 17.39% and 6.26%, respectively. Since the increase in bank lending activity was one of the intended intermediate objective of APP, we may interpret the increase in bank loans as a positive feedback of the implementation of Quantitative Easing. However, we wonder whether this increase is not too scarce against an injection of money into the banking system, that, at the end of June 2017, was equivalent to about 7% of *Monetary Financial Institutions excluding the Eurosystem* total financial assets. Concerning debt securities, the decrease in bank security holdings, observed since QE, is not surprising, considering the massive purchases of private and public securities by the Eurosystem from the banking system. About the equity market, evidence on the Quantitative Easing implemented by the Fed suggests that the decrease in the returns of bonds, generated by the purchase programmes implemented in the US, made equity investments more attractive for investors, creating a great appeal for the equity market. Despite the fact that our analysis does not allow us to observe the decline in the returns of bonds as a consequence of ECB’s QE, we can state that the decrease in euro area bank equity holdings seems to suggest that euro area *Monetary Financial Institutions excluding the Eurosystem* have not been subject to the aforementioned increased appeal for the equity market. As regards bank financial exposures via the analysed financial instruments broken down by counterparty sector, as shown in Figs. 12, 13 and 14, our estimate of growth rates of loans and deposits, debt securities, and shares and other equity held by *Monetary Financial Institutions excluding the Eurosystem* and received/issued by the different institutional sectors operating in the euro area provide us with the following findings. With particular reference to loans and deposits, as we can see from Fig. 12, since the implementation of QE, on the one hand, loans and deposits granted by*Monetary Financial Institutions excluding the Eurosystem* to i)*Monetary Financial Institutions excluding the Eurosystem*, ii)*Non-Financial Corporations*, iii)*General Governments*, and iv)*Other Financial Institutions and Non-MMF Invesment Funds* have decreased by 9.07%, 1.42%, 5.22%, and 6.81%, respectively. On the other hand, *Insurance Corporations and Pension Funds, Households,* and the*Eurosystem* have benefited from an increase in bank loans and deposits by by 3.17%, 3.51%, and 513.42%, respectively. Consequently, the empirical evidence provided by our results on loans and deposits allows us to state that there has been an increase in bank lending activity, but mostly addressed to the *Eurosystem*. Moreover, aggregated estimates on bank interactions with the financial sector and the real sector via loans and deposits provide us with empirical evidence on the increase of bank exposures to the financial sector by 14.71%, and on the slight increase of bank exposures to the real economy by 1.28%. Concerning debt securities, Fig. 13 suggests that, since the implementation of QE, only bank debt securities issued by *Non-Financial Corporations* have increased (by 14.84%), while security holdings issued by all the other institutional sectors (i.e., *General Governments*, *Monetary Financial Institutions excluding the Eurosystem*, *Other Financial Institutions*, and *Insurance Corporations and Pension Funds*), have decreased (by 14.26%, 28.63%, 11.46%, and 18.07%, respectively). Moreover, a comparison between intra-financial and financial-real exposures via securities suggests that, since QE, bank interactions with the financial sector decreased by 21.44%, while bank interactions with the real sector increased by 14.84%. Lastly, estimates on bank equity holdings by issuing counterparty sector, represented in Fig. 14, suggest that, on the one hand, bank financial exposures via equity to *Monetary Financial Institutions excluding the Eurosystem* and *Other Financial Institutions* have increased by 14.40% and 72.90%, respectively. On the other hand, bank equity holdings issued by *Non-MMF Investment Funds*, *Non-Financial Corporations*, and *Insurance Corporations and Pension Funds* have decreased by 1.11%, 22.42%, and 22.41%, respectively. Further, with reference to bank-financial interactions versus bank-real interactions via equity, it is worth mentioning that, while bank exposures to the financial sector have increased by 1.03%, bank exposures to the real sector have decreased by 22.42%. All in all, the empirical evidence coming from our investigation of the time evolution of financial linkages in the multilayer macro-network of the euro area reveals two main findings. First, since QE, there has been a comprehensive increase in the extent of bank interactions via loans and deposits, debt securities and equity with the different institutional sectors operating in the euro area. In particular, while both bank debt security and equity holdings have decreased, only bank lending activity has slightly increased. However, this increase is mainly due to the increased bank loans and deposits to the Eurosystem, rather than to the increased lending activity addressed to the real economy. Second, since QE, there has been an increase in bank exposures to the financial sector(by 3.56%) and a very slight increase in bank exposures to the real sector (by 0.55%).

### Macroeconomic effects

**Fig. 15:**
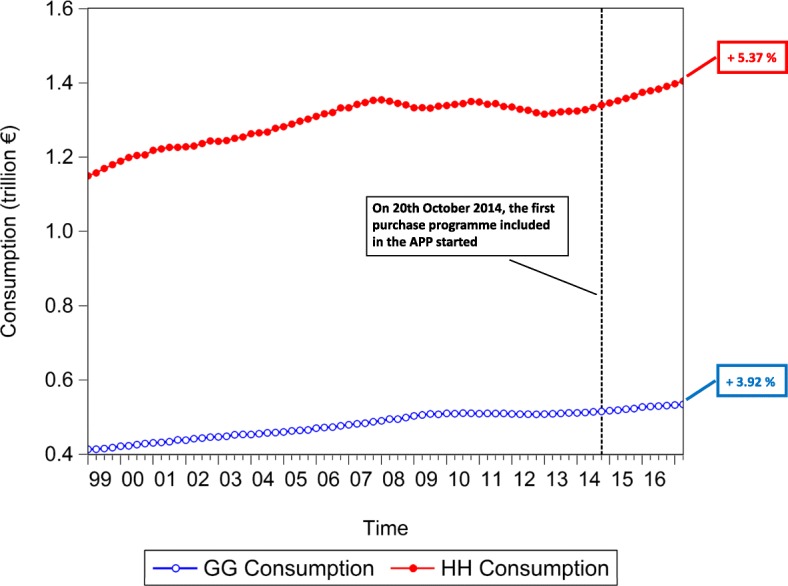
Household and General Government consumption expenditure; 1999Q1-2017Q2. Time series of the euro area household and general government consumption expenditure, over the reference period 1999Q1-2017Q2. The vertical line corresponds to the beginning of the first purchase programme (3rd Covered Bond Purchase Programme, CBPP3) included in the APP, started in 2014Q4, while the percentages on the right-hand side of the graph correspond to our estimate of the growth rate of euro area household and general government consumption expenditure, since the initiation of QE. Data source: (European Central Bank 2017b)

**Fig. 16:**
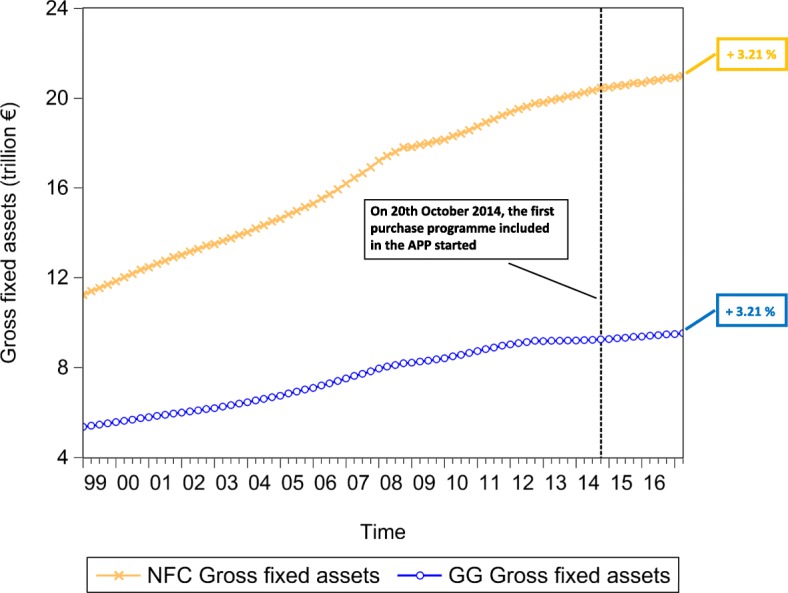
Non-Financial Corporation and General Government gross fixed assets; 1999Q1-2017Q2. Time series of the euro area non-financial corporation and general government gross fixed assets, over the reference period 1999Q1-2017Q2. The vertical line corresponds to the beginning of the first purchase programme (3rd Covered Bond Purchase Programme, CBPP3) included in the APP, started in 2014Q4, while the percentages on the right-hand side of the graph correspond to our estimate of the growth rate of euro area non-financial corporation and general government gross fixed assets, since the initiation of QE. Data source: (European Central Bank 2017b)

**Fig. 17:**
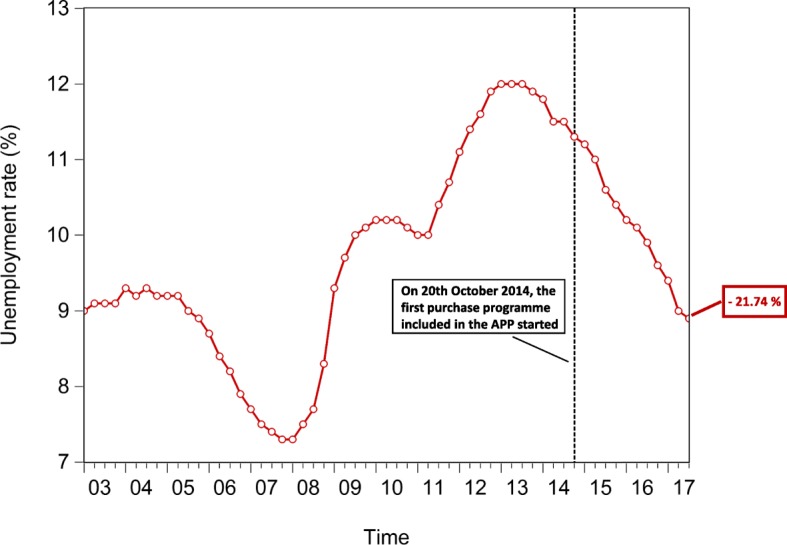
Euro area unemployment rate; 2003Q1-2017Q2. Time series of the Harmonised Unemployment Rate (HUR), over the reference period 2003Q1-2017Q2. The vertical line corresponds to the beginning of the first purchase programme (3rd Covered Bond Purchase Programme, CBPP3) included in the APP, started in 2014Q4, while the percentage on the right-hand side of the graph corresponds to our estimate of the growth rate of euro area HUR, since the initiation of QE. Data source: (Eurostat 2017)

**Fig. 18:**
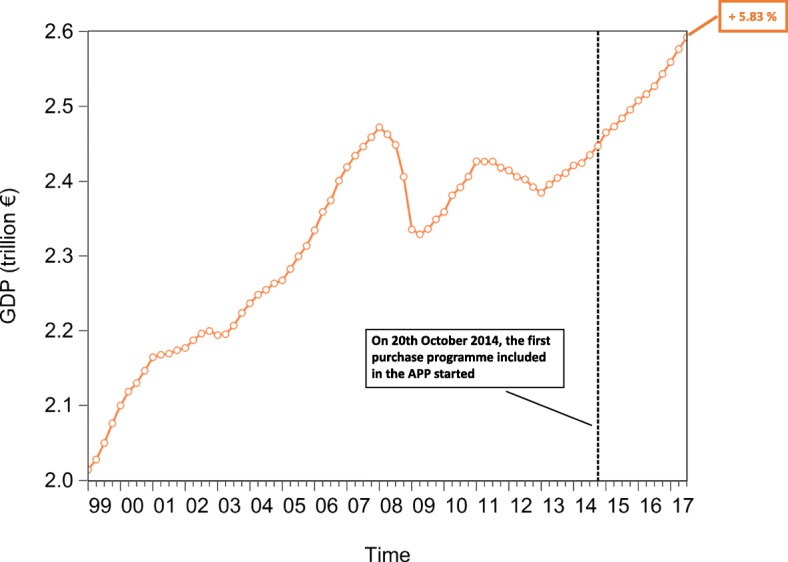
Euro area GDP; 1999Q1-2017Q2. Time series of the euro area Gross Domestic Product (GDP) at market prices, over the reference period 1999Q1-2017Q2. The vertical line corresponds to the beginning of the first purchase programme (3rd Covered Bond Purchase Programme, CBPP3) included in the APP, started in 2014Q4, while the percentage on the right-hand side of the graph corresponds to our estimate of the growth rate of euro area GDP, since the initiation of QE. Data source: (European Central Bank 2017b)

**Fig. 19:**
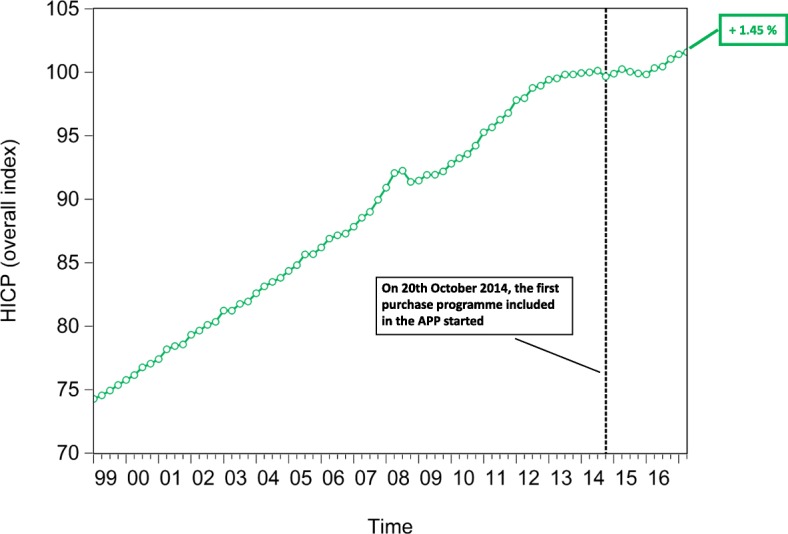
Euro area HICP; 1999Q1-2017Q2. Time series of the Harmonised Consumer Price Index (HICP), over the reference period 1999Q1-2017Q2. The vertical line corresponds to the beginning of the first purchase programme (3rd Covered Bond Purchase Programme, CBPP3) included in the APP, started in 2014Q4, while the percentage on the right-hand side of the graph corresponds to our estimate of the growth rate of euro area HICP, since the initiation of QE. Data source: (European Central Bank 2017b)

In an attempt to test the macroeconomic effects of QE, we lastly investigate the time evolution of the euro area *private and public consumption* (represented in Fig. 15), *private and public investment*, proxied by the increase in *non-financial corporation and government gross fixed assets* (represented in Fig. 16), *Harmonized Unemployment Rate (HUR)* (represented in Fig. 17), *Gross Domestic Product (GDP)* (represented in Fig. 18), and *Harmonized Consumer Price Index (HICP)* (represented in Fig. 19). Similarly to the figures included in “The dynamic perspective” section, in Figs. 15, 16, 17, 18 and 19, the vertical dashed line corresponds to the beginning of the fist purchase programme included in the APP, started in the fourth quarter of 2014. We estimated the growth rate of the aforementioned macroeconomic variables (reported in the right-hand side of Figs. 15, 16, 17, 18 and 19), since the implementation of QE. It is worth noting that, since the APP programme has been implemented, both *private and public consumption*, and *non-financial corporation and government gross fixed assets* has raised by 5.37%, 3.92%, 3.21%, and 3.21%, respectively. Further, since QE, there has been a decrease in the unemployment rate by 21.74%. In particular, the HUR among the “under 25” has dropped by 21.36%, while the HUR among the “over 25” has decreased by 21.26%.

Lastly, our data provide us with empirical evidence that both the *GDP*, and inflation have increased. In particular, *GDP* has raised by 5.83%, while the *HICP* has increased by 1.45%. Consequently, despite the macroeconomic effects of QE are probably not fully materialized yet, as the transmission mechanism may be subject to long lags (Joyce et al. 2011), our empirical evidence seems to be consistent with the chain of events included in the ECB’s narrative on QE (European Central Bank 2017c). However, our empirical analysis does not allow us to identify a causal relationship among the QE, the time evolution of financial linkages of the multilayer macro-network, and the aforementioned macro-variables.

## Conclusion

In this paper, we have conducted an empirical explorative analysis to study the implications of a specific case of unconventional policy, the ECB’s QE, in an attempt to investigate i) to what extent, the resources provided to the banking system through QE are transmitted to the real economy and ii) to what extent, the QE may alter the pattern of intra-financial interactions via loans and deposits, debt securities, and shares and other equity. Our empirical results constitute a contribution to the debate on the effectiveness of unconventional policy tools, as they reveal three main findings. First, since the implementation of QE there has been a comprehensive increase in the private banking system interactions with the euro area economy, via loans and deposits, debt securities and equity. In particular, while both bank debt security and equity holdings have decreased, only bank lending activity has slightly increased. However, this increase is mainly due to the increased bank loans and deposits to the Eurosystem, rather than to the increased lending activity addressed to the real economy. Second, since QE, there has been an increase in bank exposures to the financial sector and a very slight increase in bank exposures to the real sector. Third, since the implementation of QE, there has been an increase both in Gross Domestic Product and inflation, as well as a decrease in the level of unemployment. In a nutshell, our research has brought to light some important facts with regard to the effects of Quantitative Easing in the euro area. However, as in most serious debates, the truth lies somewhere in the middle. More specifically, on the one hand, since QE, there has not been a significant increase in the interactions between the private banking system and the real economy and, in particular, the bank-firm lending level has decreased, while the banking system is increasing its interactions with the financial sector, and, on the other hand, the overall euro area economy is experiencing growth and addressing the risk of deflation.

## Additional file

Additional file 1 Supplementary Information. (TEX 20 kb)

## Footnotes

1 Credit securitization is a process that allows an issuer to pool loans to homeowners, car buyers, students, or businesses into composite credit securities and sell them to end investors, rather than holding them to maturity on her balance sheet. Credit securitization enabled the extension of bond-based finance from governments and major corporations to a wider set of borrowers (Turner 2017).

2 Credit structuring is a practice through which the risk and the return inherent in a portfolio of loans can be divided, and allows for the creation of different tranches of credit securities, characterized by different level of risk and return (Turner 2017).

3 A collateralized Loan Obligation (CLO) is a securitiy backed by a pool of debt, generally consisting of low-rated corporate loans. A CLO grants the investor some scheduled debt payments from the underlying loans, typically associated with returns higher than average, in exchange for taking on the risk of default of the borrower.

4 A Collateralized Debt Obligation (CDO) is a structured financial product through which different assets (which serve as a collateral of the CDO) are pooled and repackaged into tranches that are separately sold to investors.

5 A Credit Default Swap (CDS) is a credit derivative via which one party buys protection against the default of a reference entity from another party. In particular, the protection buyer pays a fee to the protection seller who, in turn, promises to pay a notional amount to the protection buyer in the event that the reference entity defaults.

6 In particular, the APP, announced on January 2015, encompasses the existing i) 3rd Covered Bond Purchase Programme (CBPP3, started on 20 October 2014), and ii) Asset-Backed Securities Purchase Programme (ABSPP, started on 21 November 2014), combining them with the new iii) Public Sector Purchase Programme (PSPP, started on 9 March 2015), and iv) Corporate Sector Purchase Programme (CSPP, started on 8 June 2016) (Gambetti and Musso 2017).

7 More specifically, from March 2015 until March 2016, and from April 2017 until December 2017, the average monthly pace was€ 60 billion. From April 2016 until March 2017, the average monthly pace was€ 80 billion. From January 2018, the net purchases have been reduced to the monthly pace of€ 30 billion (European Central Bank 2017a).

8 The European Union’s Member States that have adopted the euro in stage three of Economic and Monetary Union (EMU) are Austria, Belgium, Cyprus, Estonia, Finland, France, Germany, Greece, Ireland, Italy, Latvia, Lithuania, Luxembourg, Malta, Netherlands, Portugal, Slovakia, Slovenia, and Spain.

9 Saving and loan associations include companies resembling banks in many respects, which by low must have the 65% or more of their lending in residential mortgages, though other types of lending are allowed.

10 Credit unions are alternative institutions to regular commercial banks, almost always organized as non-profit cooperatives.

11 Shadow banks could be identified as a collection of non-bank financial institutions that replicate some of the activities of regulated banks, but do not operate in the same regulatory environment.

12 Islamic banks are institutions existing to fill the need for financial services that are compliant with Islamic rules, which forbid the charging, or acceptance, of interest or other fees related to borrowing money.

13 More specifically, the purchased securities include: i) corporate sector bonds (purchased under the CBPP3 and the CSPP), ii) nominal and inflation-linked central government bonds (purchased under the PSPP), i.e., bonds where the principal and the interest payments are indexed to inflation or deflation, being contractually linked to a nationally recognized inflation measure (such as the Harmonised Index of Consumer Prices (HICP)), in order to protect investors from the negative impact of inflation, iii) bonds issued by recognized agencies, regional and local governments, international organizations and multilateral development banks located in the euro area (purchased under the PSPP), and iv) asset-backed securities (purchased under the ABSPP), i.e., financial securities collateralized (backed) by a pool of underlying assets (typically a group of illiquid assets, which are not easily tradable individually) (European Central Bank 2017a).

14 A detailed description of the financial instruments under consideration in the present analysis is provided in the Additional file 1 (Section SI2)

15 The attenuation factor has to be chosen such that it is smaller than the reciprocal of the absolute value of the largest eigenvalue of the adjacency matrix *A*.

16 The extension also allows to overcome the mathematical intractabilities connected with the fact that the presence of a cycle in the network lead to an infinite number of reverberations of the impact of a node to the others and back to itself, thus allowing a full exploitation of the linearity underlying the model, with important consequences for the analysis of the stability of the system (Battiston et al. 2016; Bardoscia et al. 2015; 2017)

17 It is worth mentioning that, thanks to the availability of new data, differently from (Perillo and Battiston 2017), we have been able to include in the analysis the institutional sectors i) *Non-MMF Investment Funds* and ii) *Rest of the World*. However, data concerning the *Rest of the World* allow us to consider this sector only from the static point of view for the purposes of our analysis.

18 Please note that all data sources in this paper have been last accessed on January 2018.

19 Unfortunately, data on insurance and pension schemes, and other accounts receivable and financial derivatives are available only at an aggregate level. Therefore, this data don’t allow us to investigate the interactions among the different institutional sectors operating in the euro area and the rest of the world via the aforementioned categories of financial instruments.

20 Unfortunately, available data on the institutional sector *Rest of the World* do not allow us to discriminate between real sector and financial sector of non-euro area countries issuing the aforementioned amount of equity held by the euro area private banking system.
